# Adult psychosocial outcomes of children with specific language impairment, pragmatic language impairment and autism

**DOI:** 10.1080/13682820802708098

**Published:** 2009-07

**Authors:** Andrew J O Whitehouse, Helen J Watt, E A Line, Dorothy V M Bishop

**Affiliations:** Department of Experimental Psychology, University of OxfordOxford, UK

**Keywords:** specific language impairment, pragmatic language impairment, autism, longitudinal, psychosocial, outcome

## Abstract

*Background:* The few studies that have tracked children with developmental language disorder to adulthood have found that these individuals experience considerable difficulties with psychosocial adjustment (for example, academic, vocational and social aptitude). Evidence that some children also develop autistic symptomatology over time has raised suggestions that developmental language disorder may be a high-functioning form of an autism spectrum disorder (ASD). It is not yet clear whether these outcomes vary between individuals with different subtypes of language impairment.

*Aims:* To compare the adult psychosocial outcomes of children with specific language impairment (SLI), pragmatic language impairment (PLI) and ASD.

*Methods & Procedures:* All participants took part in research as children. In total, there were 19 young adults with a childhood history of Specific Language Impairment (M age = 24;8), seven with PLI (M age = 22;3), 11 with high functioning ASD (M age = 21;9) and 12 adults with no history of developmental disorder (Typical; n = 12; M age = 21;6). At follow-up, participants and their parents were interviewed to elicit information about psychosocial outcomes.

*Outcomes & Results:* Participants in the SLI group were most likely to pursue vocational training and work in jobs not requiring a high level of language/literacy ability. The PLI group tended to obtain higher levels of education and work in ‘skilled’ professions. The ASD participants had lower levels of independence and more difficulty obtaining employment than the PLI and SLI participants. All groups had problems establishing social relationships, but these difficulties were most prominent in the PLI and ASD groups. A small number of participants in each group were found to experience affective disturbances. The PLI and SLI groups showed lower levels of autistic symptomatology than the ASD group.

*Conclusions & Implications:* The between-group differences in autistic symptomatology provide further evidence that SLI, PLI, and ASD are related disorders that vary along qualitative dimensions of language structure, language use and circumscribed interests. Childhood diagnosis showed some relation to adult psychosocial outcome. However, within-group variation highlights the importance of evaluating children on a case-by-case basis.

**What this paper adds**

**What is already known on this subject**

When childhood language problems persist into adulthood, they can have far reaching consequences in terms of academic, social and vocational outcomes. However, little is known about how these outcomes compare between individuals with different language profiles, for example, between those with Pragmatic Language Impairment (PLI) and those with more typical Specific Language Impairment (SLI). Previous studies have suggested that children with language impairment may develop autistic symptomatology over time, but few longitudinal studies have examined this proposal.

**What this study adds**

Adults with a childhood history of language impairment showed considerably lower levels of autistic symptomatology than adults with an autism spectrum disorder (ASD). In addition, the qualitative nature of childhood language impairment appeared to relate to adult outcome. Participants with SLI had persisting language problems and were most likely to pursue vocational training and work in jobs not requiring a high level of language/literacy ability. In comparison, the PLI group, who did not have structural language impairments, tended to obtain higher levels of education and work in ‘skilled’ professions. The SLI and PLI groups both had difficulties establishing friendships, but these difficulties were more prominent for the PLI participants. A comparison group of adults with ASD presented with considerably greater psychosocial problems. Anxiety and affective disturbances were noted in several participants.

## Introduction

There is an accumulating literature highlighting the longer-term impact of developmental language disorders. Studies indicate that many children with early communication problems present with age-appropriate language skills by the time they enter primary school (Paul *et al.*^1997^; Dale *et al.*^2003^). However, there is a significant minority whose language impairment persists beyond these years. In these children, language difficulties often become more pronounced with age, so that affected children fall further behind their typically developing peers as language demands increase during the school years (Stothard *et al.*^1998^). For example, Davison and Howlin (1997) tracked a group of children attending language units into secondary school and found a steady increase in the discrepancy between chronological age and age-equivalent scores on language tests.

Difficulties beyond the language domain also become apparent as children grow older. Children with developmental language disorders are highly susceptible to literacy problems (Snowling *et al.* 2000) as well as difficulties with broader academic abilities such as mathematical skills (Beitchman *et al.*^1996^). Unsurprisingly, these difficulties often limit academic achievement (Hall and Tomblin 1978; Aram *et al.*^1984^) and opportunities for sustained employment (Clegg *et al.*^2005^). Longer-term studies have found that those with persisting language impairments often do not attain independent living and are more likely than those without language difficulties to be the recipients of welfare benefits (Clegg *et al.*^2005^).

Social relationships also become an area of difficulty and children with language impairment often become targets of bullying during the school years (Conti-Ramsden and Botting 2004). Longer-term studies have found that social problems tend to persist beyond adolescence. Haynes and Naidoo (1991), for example, followed into early adulthood 34 ex-pupils of a residential school for children with speech and language difficulties and found that social relationships were ‘overridingly the area of greatest concern to parents’ (p. 268). Fourteen individuals in this sample rarely interacted with peers and only seven participants had experienced a romantic relationship. Clegg *et al.* (2005) reported similar findings for a sample of adults with SLI traced since childhood (*n* = 17). In their mid-30s, more than half of this sample had a limited range of friendships, while less than one-third had ever been married or in a *de facto* relationship.

An elevated prevalence of psychiatric disorders has also been noted in those with a history of language problems. Beitchman *et al.* (2001) examined the psychiatric functioning of a large sample of adults with childhood history of language problems (*n* = 77) and found that around 40% of individuals met criteria for at least one psychiatric disorder. The rate of anxiety disorders was particularly high in this sample, with around one-quarter of individuals meeting DSM-IV criteria (26.7%), compared with 8.1% of the typically developing comparison sample.

While increasingly more is known about the long-term course of developmental language disorder, little is known about the outcome of individuals with different subtypes of language impairment. Developmental language disorder is a heterogeneous category, with deficits in speech, receptive language and/or expressive language all falling under this diagnostic umbrella. The few longer-term investigations suggest that outcomes may vary depending upon the exact childhood speech/language profiles. Speech impairments, in particular, appear to be associated with better academic and psychiatric outcomes in adulthood than childhood language impairment (Beitchman *et al.*^2001^; Young *et al.*^2002^). Among those with language problems, current evidence suggests that adolescent outcomes are worse for children with global language difficulties, where there is impairment in *both* the expressive and receptive domains (Stothard *et al.* 1999); though, it is noteworthy that longer-term studies have found little association between childhood language profile and adult prognosis (Howlin *et al.* 2000).

One language phenotype that has received little attention in longitudinal studies is pragmatic language impairment (PLI). In PLI, pragmatic deficits are a dominant feature of the language profile and cannot be attributed to poor linguistic ability alone. This contrasts with the more typical form of developmental language disorder (most commonly called specific language impairment or SLI), where there is a core deficit in the structural aspects of language (morphology and/or syntax). Children with PLI are often verbose, using unusual language constructions and having difficulties with social aspects of language, such as turn-taking (Botting and Conti-Ramsden 1999). These deficits are highly similar to those observed in children with autism and there is debate as to whether PLI is best viewed as a subtype of developmental language disorder or an extension of pervasive developmental disorder (Boucher 1998). Evidence that some children have poor pragmatic language ability without additional autistic symptomatology (Botting and Conti-Ramsden 1999) has led to suggestions that PLI may fit a profile intermediate between SLI and autism (Bishop and Norbury 2002).

The current study was a follow-up investigation of individuals who participated in research conducted by Bishop throughout the 1980s and 1990s. All participants had received a clinical diagnosis of developmental language disorder and had been categorized at initial assessment as showing a profile consistent with either PLI or SLI. As part of the testing session at follow-up, the caregiver(s) of each participant completed the Autism Diagnostic Interview — Revised (Lord *et al.*^1994^), which asks for information about early childhood behaviours. Despite all participants in this study having autism discounted as a diagnosis in childhood, a small proportion of participants retrospectively met full criteria for autism (in each of the three autistic domains: communication, socialization, repetitive behaviour) according to modern diagnostic standards; a finding we have argued (Bishop *et al.*^2008^) reflects the broadening of autism diagnostic criteria from DSM-III (American Psychiatric Association, 1980) through to the most recent guidelines of DSM-IV-TR (American Psychiatric Association, 2000). For more details of this procedure, see Bishop *et al.* (2008) or Whitehouse *et al.* (present volume). The participants whose childhood behaviours retrospectively met current criteria for autism were included as a separate autism spectrum disorder (ASD) group.

A companion paper (Whitehouse *et al.* forthcoming 2007) reports that language profiles in childhood tended to persist into early adulthood, such that at follow-up the PLI group with predominantly pragmatic language deficits, the SLI group presented with considerable structural language and literacy impairments (as well as moderate difficulties with pragmatic language), and the ASD group with a combination of both structural and pragmatic language difficulties. The current study reports the psychosocial outcomes of these three groups as well as those of a comparison group, composed of adults who acted as typically developing control children in the original studies.

The first aim of this study was to determine whether childhood diagnosis relates to psychosocial outcomes. Given the persisting structural language deficits in those with SLI, we predicted that this group would be more likely to pursue vocational education and work in professions not requiring a high level of language/literacy ability (for example, manual labour). Because linguistic deficits were not a hallmark of the PLI group, we expected these individuals to be more widely spread in the level of education achieved (that is, some would attend university) and nature of profession (‘skilled’ professions as well as manual labour). However, given the impairments in social communication, we hypothesized that the PLI group would have considerable difficulty establishing friendships and romantic relationships. The surprising finding at follow-up that the SLI participants had moderate pragmatic difficulties (Whitehouse *et al.* forthcoming 2007) led us to expect that this group would also experience some difficulty with social relationships. Because the ASD group demonstrated a greater range of difficulties at follow-up (spanning both the verbal and non-verbal domains) we predicted that these participants would fare worse than the other clinical groups in terms of academic, employment and social outcome. Furthermore, while we expected the three clinical groups to all show restricted levels of independence relative to the typically developing group, this would be particularly limited in the ASD group. Finally, we expected a minority of participants to show co-morbid psychiatric impairment; however we made no prediction regarding the spread of these impairments across the three clinical groups.

The second aim of this study was to investigate whether the social impairment and repetitive behaviours that characterize autism developed over time in the participants with childhood PLI. Language impairment and autism are disorders with features and abnormalities that vary across development (Bishop and Norbury 2002). In the case of children with PLI, it is possible that deficits relating to the social and repetitive autistic domains become more apparent as children get older and are exposed to different environments and experiences. Such a finding would suggest that PLI is best viewed as a high functioning form of autism.

## Methods

### Participants

#### Childhood

Clinical participants attended special speech and language schools in the UK. Participants were subcategorized as cases of SLI or PLI based upon a series of assessments. An SLI diagnosis was given if a child scored at least 1 standard deviation (SD) below the mean on one or more standardized language assessments, but had a non-verbal IQ within normal limits. A PLI diagnosis was applied to those children who had pragmatic language difficulties that were disproportionate to their level of structural language deficit. This was determined through parent or teacher report, which was the only way of determining pragmatic impairment at the time these studies were conducted. Typically developing participants were recruited from mainstream schools, had English as a first language and had no history of developmental or neurological impairment.

#### Adulthood

At follow-up, participants were either located via the Office of National Statistics (UK), who then forwarded on information about the study (73 of 130 individuals were successfully traced: 51 language impaired and 22 typical participants), or directly approached by our research group using contact details from existing records (43 language impaired and eight typical participants). Of these individuals, 49 were assessed at follow-up. For more details of the participants, including the exact tests used in the diagnostic procedure at childhood, please refer to Whitehouse *et al.* (forthcoming 2007). This research was approved by the Central University Research Ethics Committee of Oxford University.

ADI-R interviews conducted at follow-up indicated that a number of clinical participants met current DSM-IV criteria for autism when they were children (for further information, see Bishop *et al.*^2008^). The two SLI and nine PLI participants were pooled, creating four separate groups: 19 adults with SLI (M age = 24;6, SD = 4;4, range: 16;5–31;0, five females); seven adults with PLI (M = 22;3, SD = 5;4, range: 16;2–28;9, one female), eleven adults with ASD (M = 21;9, SD = 4;0, range: 16;1–28;9, zero females) and twelve adults with no history of impairment (M = 21;7, SD = 3;2, range: 18;0–28;9; eight females). There was no difference in childhood non-verbal IQ and receptive language level between, firstly, the clinical participants who did and did not take part at follow-up, and secondly, the typically developing participants who did and did not take part at follow-up. Further analyses compared the chronological age, receptive language ability and non-verbal IQ at initial assessment of the four groups recruited at follow-up. Chronological age and non-verbal IQ did not differ between the four groups. However, the SLI, PLI, and ASD groups had significantly worse receptive language than the typical group (but did not differ between each other on this measure). See Whitehouse *et al.* (forthcoming 2007) for further details of these analyses.

### Assessments

At follow-up, the participants completed a range of psychometric assessments, while their caregivers received a standardized interview. The fourth authors, who completed the testing, have extensive experience administering standardized assessments and interviews for research purposes.

#### Autistic behaviours

The social deficits characteristic of autism were gauged with the Autism Diagnostic Observational Scales — Generic (ADOS-G; Lord *et al.*^2000^). The ADOS-G is a semi-structured assessment that uses simple activities and questions to elicit and observe the communicative and social behaviours relevant to the diagnosis of autism. Participants were administered Module 4 of the ADOS-G, which is designed for individuals with fluent language. This module relies heavily on a structured conversation, centred around topics such as day-to-day activities, social relationships and future plans. The examiner rates behaviours as they occur according to a numerical scale of symptom severity, with a score of zero indicating no observed abnormality. The assessment was given by one of two examiners (EL and AW), both of whom were certified as having achieved acceptable levels of reliability for this module. Twelve of the ADOS-G assessments administered by EL and twelve administered by AW (50% of all ADOS-G assessments conducted) were cross-examined by the other researcher (blind to the others' ratings). An acceptable level of reliability was achieved (85%). Where there were discrepancies, the researchers discussed the participants' responses until agreement was made.

Because the ADOS-G provides insufficient opportunity to observe restricted and repetitive interests, information relating these behaviours was obtained from the ‘current functioning’ section of the Autism Diagnostic Interview — Revised (ADI-R; Lord *et al.*^1994^) The ADI-R is a structured interview conducted with the person who was the participants' principal caregiver when they were a child. The interviewee is required to give detailed descriptions of the child's behaviours, which the interviewer then codes into a numerical scale of symptom severity (zero indicating no observed abnormality). The interview typically takes around 3 h and was administered by one of three trained examiners (DB, HW and AW).

#### Other outcome measures

Parents completed a short questionnaire designed by our research group to elicit additional information about the participants' educational and employment background. Parents were also asked about the level of independence achieved by the participants, including self-care activities, travel and financial management. Information relating to friendships was obtained from the ‘current functioning’ section of the ADI-R (question 65, current behaviour), while the ADOS-G provided information about romantic relationships. To check for the veracity of self-report, parents were also asked to report on the participants' romantic relationships. Where there was discrepancy between participant and parent report, the responses were examined to determine the most accurate answer, taking into account factors such as participant level of functioning and parental insight. Generally speaking, participant report was favoured for high functioning participants (that is, those with a full array of independent behaviours), while parent report was favoured for low functioning participants.

Parents were also asked for information concerning the participants' psychiatric history. In the case that a participant had been referred to a psychiatrist, information was obtained about the purpose and outcome of this referral.

## Results

### Social outcome

#### Friendships

Information relating to friendship quality was obtained from the ‘current functioning section’ of the ADI-R (Table 1). For the three participants without ADI-R data (SLI = 2; PLI = 1), this information was derived from the ADOS-G and recoded to fit the ADI-R parameters. All of the participants in the Typical group were considered to have at least one close friendship. In comparison, a substantial proportion of participants in the SLI (21.1%) and PLI groups (57.1%) and all of the participants in the ASD group did not have any close friendships. Mann–Whitney *U*-test comparisons^1^ confirmed that the ASD and PLI groups had significantly poorer quality of friendships than the Typical group (ASD versus Typical: (*U* = 0, *z* = −4.41, *p*<0.01; PLI versus Typical: *U* = 18, *z* = −2.85, *p*<0.01), while the comparison between the SLI and Typical group fell just short of significance (*U* = 90, *z* = −1.67, *p* = 0.09). Further analyses revealed that the ASD group had significantly poorer quality of friendships than the SLI group (*U* = 16, *z* = −4.1, *p*<0.01). There was also a trend for the PLI group to have greater impairment than the SLI group (*U* = 41.5, *z* = −1.77, *p* = 0.08).

**Table 1:** Friendships and romantic relationships experienced by the four groups

	SLI	PLI	ASD	Typical
*Friendships*^a^				
Normal	15 (78.9)	3 (42.9)	0 (–)	12 (100)
Limited	2 (10.5)	2 (28.6)	4 (36.4)	0 (–)
Acquaintances	2 (10.5)	1 (14.3)	4 (36.4)	0 (–)
None	0 (–)	1 (14.3)	3 (27.3)	0 (–)
*Romantic relationships*^b^				
Romantic relationship (greater than or equal to 3 months)	8 (53.3)	2 (50)	0 (–)	6 (100)
No romantic relationship	7 (47.7)	2 (50)	5 (100)	0 (–)

Notes: Date shown are the number (and proportion) of participants in each group.

Normal: has one or more friends of roughly their own age with whom they share a variety of interests and social activities.

Limited: has one or more ‘friends’ with whom they meet to share their interests outside of a prearranged setting, but limited in terms of restricted interests and responsiveness/reciprocity.

Acquaintances: people with whom the subject has some kind of personal relationship involving seeking contact, but only in a group situation or school/work.

None: no peer relationships that involve selectivity or sharing.

a SLI = 19; PLI = 7; ASD = 11; Typical = 12.

b SLI = 15; PLI = 4; ASD = 5; Typical = 6.

#### Romantic relationships

To avoid unfairly penalizing younger participants who may not have had the opportunity to develop a romantic relationship, participants were only considered in this analysis if they were 21 years or older (excluding four participants from the SLI group, three from the PLI group and six each from the ASD and Typical groups). Table 1 shows that all six Typical participants, two of the four PLI participants and eight of the 15 SLI participants had experienced a romantic relationship of at least three months duration. Five participants in the SLI group had children, four of whom (all men) were married to their children's mother. No PLI participant was married or had children. All reported romantic relationships were heterosexual. In contrast, none of the five ASD participants had experienced a romantic relationship.

### Academic

When tested in childhood, all of the clinical participants were either attending schools that specialize in the education of children with speech and language disorder or special language units attached to mainstream schools. All participants recruited in secondary school (SLI = 8, PLI = 1, ASD = 3) attended speech and language schools until at least 16 years of age. There was some variability in the secondary schools attended by those participants recruited in primary school (SLI = 11; PLI = 6, ASD = 8). The majority of participants attended either a speech and language school (SLI = 6, PLI = 2, ASD = 4), a special language unit attached to a mainstream school (ASD = 2) or a mainstream school with additional educational support (usually in the form of a teacher's assistant; SLI = 3, PLI = 3). Two ASD participants recruited in primary school attended a secondary school for children with autism and one participant in the SLI group did not have any formal schooling beyond 14 years. One member each of the PLI and SLI groups attended mainstream school without extra educational support.

Between the ages of 14 and 16, children in the UK work towards achieving their Graduate Certificate of School Education (GCSE). Students are eligible to take ‘Advanced levels’ of study (A-Levels; taken between the ages of 16–18 and typically required for entrance to university) if they achieve five or more passes at GCSE level. Vocational colleges are an alternative route for further education and provide qualifications (National Vocational Qualifications or NVQs; Business and Technology Education Council or BTEC) for job-specific skills (for example, hairdressing). Five participants were still of school age (≤18 years) and were examined separately. Three of these participants were working towards vocational qualifications (SLI = 1, PLI = 1, ASD = 1), while another PLI participant had achieved six passes at GCSE level and was studying for A-Levels. A final (ASD) participant did not study for GCSE and had no plans to undertake further education.

There were 18 SLI, five PLI and nine ASD participants who were older than 18 years. Over half of the participants in each of these groups did not gain five or more passes at GCSE level (SLI = 11, PLI = 3, ASD = 9). However, the majority of these individuals achieved some form of vocational qualification (for example, welding, catering, horticulture, tourism); three participants only had no formal qualification (SLI = 2, ASD = 1). Three ASD participants and two SLI participants attended colleges for individuals with special needs. Two participants (ASD = 1, PLI = 1) who did not achieve five or more passes at GSCE level, completed a bridging course at a vocational college, which allowed them to transfer to university.

Of the clinical participants who were eligible to take A-Levels (SLI = 7, PLI = 2, ASD = 1), three in the SLI group and one in the ASD group elected to study for vocational qualifications (SLI: painting/decorating, retail, business; ASD: retail). Three participants the SLI group, two in the PLI group and one in the ASD group took A-Levels. Two PLI participants had completed university degrees (computer science, nursing) at the time of assessment, while one ASD participant and three SLI participants were mid-way through their university degrees (ASD: design; SLI: history, computer science X 2). In comparison, all of the Typical participants took A-Levels and two-thirds of the participants went on to study for a university degree (for example, history, occupational therapy, business management, mechanical engineering).

### Employment

Employment history was then examined between groups. A considerable number of participants in each group were still in further education (SLI = 6, PLI = 4, ASD = 5, Typical = 8) and were excluded from examination.^2^ Seven of the remaining 13 SLI participants had been in continuous full-time employment, while a further four had experienced at least one period of full-time employment (greater than 3 months). All employed SLI participants were either in manual labour (carpet fitter, cleaner, painter) or service professions (for example, receptionist). All three PLI participants not in further education had been continuously employed in ‘skilled’ professions since beginning work (nurse, website designer, computer software designer). Two participants in the ASD group had been in continuous full-time employment (both factory workers), while three ASD participants could manage part-time work only (two were cleaners in a hotel and the other was a cleaner in a shopping centre). Two members of the SLI group (ages = 28;2 and 23;2) and one member of the ASD group (22;8) had never been employed. Three of the four typical participants had been in continuous full-time employment (researcher, office manager and kitchen porter), while the other participant had experienced periods of unemployment (at time of testing, employed as a hospital administrator).

### Independence

All participants were able to cope with self care activities such as bathing, getting dressed and preparing/buying food. Similarly, all but one participant in each of the PLI and SLI groups and four in the ASD group could use the telephone without any difficulty. While the majority of participants in each group could travel short distances to familiar locations using a bicycle, public transportation or by driving, six participants in the ASD group and two participants in each of the SLI and PLI groups had difficulty travelling independently to unfamiliar locations. Similarly, while nearly all participants had a bank account, five SLI, two PLI and eight ASD participants were unable to manage this independently. All participants in the typical group had achieved these markers of independence.

Mann–Whitney *U*-tests were conducted to examine group-differences in independence.^3^ All clinical groups were found to be less independent than the Typical group (for all comparisons, *p*<0.02). The ASD group was found to be less independent than the other two clinical groups (SLI versus ASD: *U* = 32, *z* = −3.35, *p*<0.001; PLI versus ASD: *U* = 11, *z* = −2.65, *p*<0.05).

### Psychiatric problems

A number of participants in each group had received a psychiatric referral (SLI = 5, PLI = 1; ASD = 4; Typical = 2). Five SLI participants had been diagnosed with major depressive disorder, three of whom had a comorbid anxiety disorder (obsessive compulsive disorder, social phobia, agoraphobia). Two of the three participants with depression and anxiety disorder had spent a period of time in a psychiatric hospital (one for two weeks and the other for two months). The other SLI participant with this comorbidity was reported to be violent towards family members and had a history of suicide attempts, the first of which occurred when he was 10 years of age. Three ASD participants had received a diagnosis of major depressive disorder and comorbid anxiety disorder (all three, generalized anxiety disorder), while another ASD participant had been diagnosed with obsessive compulsive disorder. One PLI participant had a history of violence towards his parents and had been diagnosed with general anxiety disorder. This participant (who has a paternal history of schizophrenia) had also experienced a psychotic episode in early adolescence where he had visions of religious figures. Two participants in the typical group had received a diagnosis of major depressive disorder.

### Autistic behaviours

#### Social impairment

Table 2 shows the number of participants in each group who rated on ADOS-G items relating to social impairment (that is, those who did not score a zero). One participant in the SLI group was not administered the ADOS-G because of the onset of mental illness and was omitted from this analysis. Few participants demonstrated unusual eye contact and there was no group difference for this item. The ASD group scored prominently on the remaining items, indicating high levels of social impairment. A considerably smaller number of participants in the SLI group were coded as abnormal and Chi-square (χ^2^) analyses indicated a number of significant differences between the SLI and ASD groups. Over half of the participants in the PLI group had an inappropriate range of facial expressions, which was a proportionally similar number of participants to the ASD group. Two of the six PLI participants rated on items relating to empathy, shared enjoyment in interaction, communication of own affect and the amount of reciprocal social communication; a significantly greater proportion of impairment than that observed in the typical group. However the level of deficit in the PLI group was considerably less than the ASD group, and there were significant differences between the groups on the majority of items. There were seven SLI and three PLI participants who rated on none of the items, while all ASD participants rated on at least three of the items listed in Table 2.

**Table 2:** Number (and proportion) of participants in the three clinical groups rated as abnormal on ADOS-G items relating to social skills

Item	SLI (*n* = 18)	PLI (*n* = 7)	ASD (*n* = 11)	Typical (*n* = 12)	Statistically significant differences
Unusual eye contact	2 (11.1)	2 (28.6)	4 (36.4)	0 (–)	Typical<(PLI = ASD)
Facial expressions directed to others	5 (27.8)	4 (57.1)	9 (81.8)	0 (–)	Typical<SLI<(PLI = ASD)
Empathy/comments on others' emotions	6 (33.3)	2 (28.6)	11 (100)	0 (–)	Typical<(SLI = PLI)<ASD
Shared enjoyment in interaction	2 (11.1)	2 (28.6)	7 (63.6)	0 (–)	Typical<PLI<ASD; SLI<ASD
Communication of one's own affect	3 (16.7)	2 (28.6)	7 (63.6)	0 (–)	Typical<PLI<ASD; SLI<ASD
Insight into the nature of social relationships	4 (22.2	1 (14.3)	10 (90.9)	0 (–)	(Typical = SLI = PLI)<ASD
Quality of social overtures	2 (11.1)	1 (14.3)	9 (81.8)	0 (–)	(Typical = SLI = PLI)<ASD
Quality of social response	6 (33.3)	3 (42.9)	10 (90.9)	0 (–)	Typical<(SLI = PLI)<ASD
Amount of reciprocal social communication	3 (16.7)	2 (28.6)	8 (72.7)	0 (–)	Typical<PLI<ASD; SLI<ASD
Quality of rapport	3 (16.7)	1 (14.3)	7 (63.6)	0 (–)	(Typical = SLI = PLI)<ASD

Note: Only statistically significant differences are presented.

#### Stereotyped and repetitive behaviours

The number of participants in each group reported to have current abnormalities on ADI-R items relating to stereotyped behaviours and repetitive behaviours (that is, those who did not score a zero) are shown in Table 3. Three parents did not complete the ADI-R (one set of parents could not be contacted and two refused to participate). While, overall, there were few participants who rated on any items, this was especially true for the SLI and Typical groups. Chi-square analyses indicated a significantly greater prevalence of circumscribed interests, verbal rituals and unusual sensory interest amongst the ASD group relative to the SLI group. Circumscribed interests were found in two of the six PLI participants, which was a significantly greater proportion than that reported in the SLI and Typical groups. One of these PLI participants was also reported to have hand and finger mannerisms, stereotyped body movements and unusual sensory interests. No other PLI participant had stereotyped or repetitive behaviours.

**Table 3:** Number (and proportion) of participants in each group showing stereotyped and repetitive behaviours according to the ADI-R

Item	SLI (*n* = 16)	PLI (*n* = 6)	ASD (*n* = 11)	Typical (*n* = 12)	Statistically significant differences
Unusual preoccupations	1 (6.2)	0 (–)	1 (9.1)	0 (–)	No differences
Circumscribed interests	0 (–)	2 (33.3)	4 (36.4)	0 (–)	(Typical = SLI)<(PLI = ASD)
Verbal rituals	0 (–)	0 (–)	4 (36.4)	0 (–)	(Typical = SLI)<ASD
Compulsions/rituals	1 (6.2)	0 (–)	1 (9.1)	0 (–)	No differences
Hand and finger mannerisms	1 (6.2)	1 (16.7)	2 (18.2)	0 (–)	No differences
Stereotypical body movements	1 (6.2)	1 (16.7)	1 (9.1)	0 (–)	No differences
Repetitive use of objects	1 (6.2)	0 (–)	3 (27.3)	0 (–)	Typical<ASD
Unusual sensory interests	0 (–)	1 (16.7)	4 (36.4)	0 (–)	(Typical = SLI)<ASD

Note: Only statistically significant differences are presented.

## Discussion

There is a current dearth of data on the long-term outcomes of children with developmental language disorders. Existing longitudinal studies in this area have grouped together children with a wide range of language profiles (for example, Clegg *et al.*^2005^; and Haynes and Naidoo 1991), making it difficult to determine whether outcomes vary between different subtypes of language impairment. The first aim of this study was to compare the psychosocial outcomes in adulthood of children diagnosed with two subtypes of language disorder: SLI and PLI. A third group composed of adults with ASD acted as a comparison group. The findings of the current study highlighted a number of differences between the SLI, PLI, and ASD groups and, in conjunction with the findings reported in Whitehouse *et al.* (forthcoming 2007), allowed us to build a picture of ‘typical’ adult outcomes of children with these diagnoses. The Appendix contains a case study from each group. These particular case studies were chosen because their non-verbal IQ and receptive language ability at T1 most closely resembled the means of their respective groups at this time. Here, we provide a summary of the ‘typical’ outcome of each group.

The children with a history of SLI presented with considerable structural language and literacy deficits at follow-up as well as moderate difficulties with pragmatic language. The individuals were not academic high-achievers but most had gained some form of vocational qualification. There were some difficulties in achieving consistent employment and those who were employed worked predominantly in professions that did not demand high language and literacy levels (for example, manual labour). A significant minority of individuals with SLI found establishing and maintaining friendships very difficult, and romantic relationships also proved challenging for the SLI group. However, the vast majority of SLI participants showed a variety of independent behaviours. The PLI group presented with considerably better structural language skills than the SLI group but had persisting difficulties with language use. The PLI group appeared more academically able than the SLI group and tended to work in ‘skilled’ professions. Despite the vast majority of PLI participants demonstrating a range of independent behaviours, few had experienced close friendships or romantic relationships. The ASD group was found to have substantial pragmatic difficulties in addition to some problems with structural language (for example, vocabulary, and verbal short-term memory) and literacy (reading). Social impairment and repetitive behaviours were also found to persist in these individuals. Most individuals had gained or were working towards some form of vocational qualification, but stable employment proved to be an area of difficulty for this group. The ASD participants were reported to show some independent behaviours, but had particular difficulty with travelling to an unknown destination and managing their own finances. Poor social relationships were perhaps the most striking characteristic of this group, with parent report suggesting that no ASD participant had close friendships. These findings are largely consistent with previous studies of the adult outcome of children with autism (for a review, see Howlin 2000).

Although the group sizes were small, the unique data collected in this study allow us to draw some tentative conclusions regarding the validity of forecasting adult outcomes from the pattern of language impairment in childhood. The differences in outcome for the SLI and PLI groups, particularly with regards to academic achievement (SLI<PLI) and social aptitude (SLI>PLI), are in line with what would be expected from the two groups' language profile in childhood. While adequate structural language and literacy abilities are crucial to higher education, pragmatic difficulties do not necessarily limit achievement in this area. On the other hand, problems with the pragmatic aspects of language are likely to be a greater obstacle to establishing and maintaining social relationships than difficulties with structural language (Howlin *et al.* 2000). Despite the relatively small number of participants who agreed to take part at follow-up, these findings provide clear evidence that the qualitative nature of language impairment in childhood bears influence on psychosocial outcomes in adulthood. We would, however, like to stress that there remains variability of outcome within these diagnostic categories. Broad diagnostic subtypes are able to provide some indication of future outcomes; however we suggest that more accurate predictions are likely to be made by obtaining a detailed appreciation of individuals' strengths and difficulties on a case-by-case basis.

The second aim of this study was to investigate whether autistic symptomatology developed over time in the children with PLI. The striking similarity in pragmatic deficits observed in children with PLI and autism has stimulated debate as to whether the two disorders are variable expressions of the same disorder (Boucher 1998). Recent evidence that children with PLI do not show the same degree of social deficit and repetitive behaviours that characterize autism supports an alternative view that a diagnostic distinction should be maintained between the two disorders (Bishop and Norbury 2002). However, autistic symptomatology can vary with age and no study has tracked the longitudinal course of PLI. Direct observation at follow-up found that a small number of PLI participants experienced social difficulties similar to those seen in the individuals with ASD. The prevalence of these problems was often equivalent to that observed in SLI group and considerably reduced compared to the ASD group, in which almost all participants had significant social impairment. A low proportion of individuals in the PLI group were found to display stereotyped and repetitive behaviours; two of the six participants with ADI-R data were reported to have circumscribed interests, and one of these participants was also reported to have hand and finger mannerisms, stereotyped body movements and unusual sensory interests at follow-up. However, once again, the frequency of these behaviours was considerably reduced compared to the ASD group. Together, these findings indicate that autistic symptomatology does not develop over time in all individuals with primary pragmatic difficulties, and provides further caution against treating PLI as equivalent to ASD.

Importantly, however, these adult data corroborate the findings of childhood investigations that have shown no clear diagnostic boundary between SLI and PLI on one hand, and PLI and autism on the other (Bishop and Norbury 2002). Although the ‘typical’ profile of the three groups were somewhat distinctive in terms of structural language impairment, pragmatic language difficulties and restricted interests, there were at least some participants in each group who showed a pattern of impairment that was not characteristic for their diagnosis. For example, a considerable proportion of SLI participants had considerable pragmatic difficulties, while three PLI participants presented with significant linguistic impairment (Whitehouse *et al.* forthcoming 2007). Similarly, a number of PLI participants demonstrated low levels of the social difficulties and stereotyped behaviours that are characteristic of autism. These findings support a dimensional approach to communication disorders, in which individuals can show different combinations and severity of impairment in language structure, social use of language and restricted interests (Bishop, 2000).

Another important finding that replicated previous research (Beitchman *et al.*^2001^) was of the relatively high prevalence of psychiatric disorders reported among the clinical participants. Major Depressive Disorder was particularly common in the sample, being reported in five of the 19 participants in the SLI group (26.3%) and three of eleven ASD participants (27.3%). In all cases, these disturbances were severe enough to warrant a psychiatric referral and, in two SLI cases, require hospitalization. This compares with two of 11 participants in the Typical group (18.2%) and a lifetime prevalence in the general population of around 16% (Kessler *et al.*^2003^). Anxiety disorders were also relatively common, with three participants in each of the SLI (15.7%) and ASD groups (27.3%) having a disorder under this diagnostic umbrella. Clearly, the long-term mental health of individuals with developmental disorders is an important issue and identifying potential causes of psychiatric difficulties needs to be made a priority of future research. Social, cognitive and genetic risk factors are all known to influence the onset of affective disturbances (Lau *et al.*^2007^) and research at all three ‘levels’ will converge on a better understanding of these problems in individuals with communication and pervasive developmental disorders.

One point that we would like to emphasize relates to the possibility of participant bias. The participants in the current study were drawn from a large sample of individuals who took part in studies as children. Although the participants who agreed to take part at follow-up did not differ from the remainder of the sample in terms of receptive language and non-verbal IQ (Whitehouse *et al.* forthcoming 2007), it is plausible that they did differ in terms of psychosocial outcomes. For example, participants with lower levels of literacy, educational achievement and social independence may have been less likely to participate because (1) they found it difficult to understand the information package that was used for recruitment at follow-up, or (2) the study would highlight what they may perceive as a relatively poor outcome. Conversely, it is possible that participants with high support needs were more likely to participate, owing to their (or their caregiver's) desire to report on the difficulties they currently experience. We took some precautions to minimize these risks, such as the inclusion of an information DVD, which reduced the demands on literacy and also familiarized the potential participants with the research team and procedure. However, beyond these precautions, it is impossible to determine exactly how far the outcome of the current participants is representative of those who declined to participate. Studies that systematically track cohorts of children with language impairment over time (for example, the Manchester Language Study; Conti-Ramsden *et al.*^2001^) are likely to not only incur lower levels of sample attrition, but also provide useful information on those participants who decline to take part at a later date.

However, what is clear from this study is that language impairment often persists to adulthood and has wide-ranging implications for broader life outcomes. The findings highlight the pressing need for ongoing intervention for these individuals that focus not only on important language and literacy skills, but also on strategies that will promote psychosocial adjustment.

## Notes

1 Subjects were assigned a rank of zero for one or more close friendships, a score of 1 for limited friendships, a score of 2 for acquaintances only, and a score of 3 for no peer relationships.

2 All participants attending special colleges (SLI = 2, ASD = 3) were involved in ‘guided employment’ programmes, in which individuals undertake unskilled and often unpaid work in a supported environment.

3 A rating of zero was given to participants who were able to cope with self-care activities, travel independently to familiar and unfamiliar places, and manage their own finances without help; a rating of 1 was given if some help was required in these areas; and a rating of 2 was given if significant help was required in these areas.

## Appendix: Case study from the SLI, PLI, and ASD groups

Some specifics have been altered to preserve anonymity.

### SLI (participant SLI15)

This participant was reported by his mother to have had good language comprehension as a child but extreme difficulty in expressing himself. He received a speech and language assessment at age 5;6, which recommended he attend a boarding school for children with speech and language difficulties (where he remained for the entirety of his schooling). At initial assessment (age 10;6), he had a non-verbal IQ of 94 with a receptive and expressive language standard score of 83 and 54, respectively (standard score mean = 100, SD = 15). At follow-up (age 30;0), his non-verbal IQ was 104. He had persisting deficits in vocabulary and receptive grammar (both at least 1 SD below the mean) and demonstrated numerous articulation errors. He also had very poor literacy ability, scoring close to floor on the tests of reading and spelling. He did not sit for GCSEs but attended a vocational college where he took a course in catering (passing with distinctions). Currently, he works in a café as a cook. Although he lives with his parents, he pays them rent and is financially independent. He described one close friend who has recently moved away and says that his language impairment makes it difficult for him to establish new social relationships. He has never had a girlfriend (citing ‘no one wants me’) but expressed a strong desire to one day get married. In his spare time he likes to play computer games and go to the cinema. His mother described him as ‘polite, well mannered and very helpful’, but ‘down on confidence’ and in need of more close friends.

### PLI (PLI03)

Parents reported that this participant was slow in learning vocabulary as a child and that he would use a standard set of phrases ‘that were picked up from other people’. As a child, he was also reported to be ‘self-centred in his choice of conversation topic’ and to get confused by his over-literal interpretations of other people's language. At initial assessment, he had a non-verbal IQ of 115, an expressive language score within normal range and a receptive language score that bordered the lower limits of the normal range. When reassessed in adulthood (26;9), his non-verbal IQ was 118. He demonstrated no difficulty with any of the structural language and literacy tasks, but had a structural–pragmatic mismatch composite of −14 on the Communication Checklist — Adult (Whitehouse and Bishop forthcoming), indicating considerable levels of pragmatic language difficulty. The ADOS-G elicited few social deficits characteristic of autism, while the ADI-R indicated an absence of both restricted interests and repetitive behaviours. He attended a residential speech and language school for primary and secondary education, after which he transferred to a vocational college and then to university, where he completed a degree in Business Information Systems with Honours. He currently works as a computer software designer. His parents report that he still used stereotyped phrases and that it is often difficult to stop him talking. Parent report and direct observation also indicated very infrequent use of gesture in conversation. He sees two friends regularly, but his parents reported that he has had to learn to modify his ‘inappropriateness’ with these individuals.

### ASD (ASD06)

Originally diagnosed with developmental language disorder, this participant retrospectively met criteria for autism according to the ADI-R. His ADI-R scores were 14 (autism cut-off = 10), 14 (autism cut-off = 10), and 4 (autism cut-off = 3) for social interaction, communication and repetitive behaviours respectively. He said his first words around age 3 and had little to-and-fro chat during primary school. At age 6 he had a non-verbal IQ of 105, a receptive language standard score of 68 and an expressive language standard score of 65. At follow-up (20;5), he had a non-verbal IQ of 106 and performed within normal limits on tests of articulation, receptive grammar, production of connected language, and spelling. However, this participant had a limited vocabulary (age-equivalent of 11 years) as well as some degree of difficulty with reading and verbal short-term memory (around 1 SD below mean). Pragmatic impairment was the most prominent feature of his language profile, with a structural–pragmatic mismatch of −9 on the Checklist Communication — Adult (Whitehouse and Bishop forthcoming). He rated on all ADOS-G items relating to social ability, but no ADI-R ‘current functioning’ items describing repetitive behaviours. He attended a special language school until age 16 years, leaving without undertaking any GCSEs, and then completed a one-year guided employment scheme at a special-needs vocational college. He currently has paid work for four hours a week as a cleaner in a cinema and remains financially dependent on his mother. Social relationships are an area of great concern for his mother, who described his friendships as ‘acquaintances at best’. Despite presenting with noticeably flat affect, this participant had never been referred to a psychiatrist for depressive illness.
